# Association of an integrated management strategy with operating room efficiency, turnover time, and satisfaction: a retrospective before-after study

**DOI:** 10.3389/frhs.2026.1796885

**Published:** 2026-06-26

**Authors:** Shushu Zhong, Shanshan Du

**Affiliations:** The Second People's Hospital of Lianyungang, Lianyungang, China

**Keywords:** information technology, operating room turnover time, process optimization, retrospective before-after study, team collaboration

## Abstract

**Background:**

This study evaluated the association between implementation of an integrated management strategy—combining process optimization, information technology, and team collaboration—and changes in operating room turnover time and efficiency.

**Methods:**

This retrospective before-after study analyzed 120 elective non-first surgeries [60 pre-implementation (February–July 2024) and 60 post-implementation (August 2024–February 2025)] across five surgical departments at a tertiary hospital. On August 1, 2024, hospital administration introduced a new management protocol; the research team played no role in its design or implementation. Outcomes included turnover time, operating room utilization, preoperative readiness, and patient and surgeon satisfaction.

**Results:**

The post-implementation cohort showed shorter turnover time (40.75 ± 8.33 vs. 52.75 ± 8.20 min; *Δ* = –12.00 min, 22.7%), higher operating room utilization (82.33% vs. 71.91%; *Δ* =  + 10.42 percentage points), improved preoperative preparedness (87.61 ± 6.22 vs. 81.38 ± 4.69; *Δ* =  + 6.23 points), and higher patient satisfaction (39.05 ± 2.42 vs. 35.32 ± 2.42; *Δ* =  + 3.73 points) and surgeon satisfaction (31.42 ± 3.08 vs. 26.03 ± 2.41; *Δ* =  + 5.39 points).

**Conclusion:**

In this retrospective before-after study, implementation of the integrated management strategy was associated with shorter turnover time, higher operating room utilization, and improved satisfaction among both patients and surgeons. However, given the absence of a concurrent control group, causal inference is limited. These findings suggest a potential association worthy of further evaluation in prospective controlled studies.

## Background

With an annual global increase of 5.3% in surgical volume and the widespread adoption of minimally invasive techniques, perioperative management in healthcare institutions faces growing challenges (1, 2). The imbalance between supply and demand for operating room resources has become increasingly pronounced. Efficient sequential surgery—performing consecutive procedures in the same operating room—has emerged as an essential strategy to enhance resource utilization and is now a core component of modern operating room management. Successful implementation of this model depends not only on technical proficiency but also on improved timeliness across multiple processes, including patient transfer, instrument preparation, and environmental disinfection.

Studies indicate that inefficiencies in operating room management prolong room occupancy by 15%–22%, leading to extended turnover times and reduced overall utilization (3, 4).These inefficiencies directly increase operational costs and negatively impact hospital performance Furthermore, suboptimal operating room workflows are associated with increased safety risks, including a higher incidence of adverse events (5). Prolonged preoperative waiting times also contribute to heightened patient anxiety, further diminishing satisfaction (6). Consequently, improving operating room efficiency has become critical for ensuring patient safety, optimizing resource allocation, and enhancing satisfaction outcomes. Among key performance indicators, turnover time —the interval between the completion of one surgery and the start of the next in the same room—plays a pivotal role. Prolonged turnover times initiate a cascade effect: each additional 10 min is associated with a 7.2% decrease in operating room utilization (7). These challenges have given rise to a vicious cycle of “rising service demand coupled with declining operational efficiency,” highlighting the limitations of traditional management models in coordinating complex surgical schedules and multidisciplinary teamwork, particularly in cross-departmental collaboration and dynamic resource allocation (8).

Evidence suggests that process optimization can eliminate redundant steps, reducing average turnover time by 19 min and decreasing operating room delay rates by over 44%. These improvements translate into annual cost savings of $169,597 in operating room operations and $88,939 in sterile processing department (SPD) expenses (9). Furthermore, information technology–enabled management, such as the use of electronic dashboards and real-time scheduling systems, has been shown to reduce communication delays by 44% (10). Concurrently, a team-based, multidisciplinary collaboration model has increased surgeon satisfaction by 32% (11).

However, existing evidence primarily focuses on isolated interventions, with limited evaluation of the synergistic effects of integrating process reengineering, technological enablement, and team coordination. Therefore, developing and validating a comprehensive management strategy that combines process optimization, information technology support, and enhanced team collaboration holds significant practical value for improving operating room efficiency, ensuring patient safety, and enhancing hospital operational performance. This retrospective study evaluates the association between implementation of an integrated ‘Process-Information-Team’ management strategy and changes in OR turnover time, efficiency, and stakeholder satisfaction.

## Materials and methods

### Study design

This retrospective before-after study was conducted at a tertiary hospital in China, comparing operating room performance between two historical cohorts defined by a hospital-wide policy change implemented on August 1, 2024, as part of a routine quality improvement initiative, in which the research team played no role in designing, selecting, or implementing the protocol. Patients were assigned to exposure groups solely based on the temporal relationship between their surgery date and the policy change: the pre-implementation cohort (unexposed) included patients undergoing elective non-first surgeries between February 1, 2024, and July 31, 2024 (*n* = 60) who received care under the prior management protocol, while the post-implementation cohort (exposed) included those undergoing similar surgeries between August 1, 2024, and February 28, 2025 (*n* = 60) who received care under the new protocol. No concurrent control group was available, and no randomization was performed. This design is analogous to a natural experiment in that the exposure was determined by the timing of a policy change rather than by investigator assignment, though no randomization was performed. The study was approved by the hospital's Ethics Committee (approval no. 2025K066) on May 6, 2025, prior to data extraction, and informed consent was waived due to the retrospective, anonymized nature of data analysis using pre-existing routine records.

### Study population and sampling

From 486 eligible elective non-first surgeries performed between February 2024 and February 2025 across five surgical departments (general surgery, orthopedics, obstetrics and gynecology, urology, and thoracic surgery), we selected 120 cases for analysis. To ensure balanced representation across specialties and to reduce selection bias, we employed systematic sampling within each department: from each department, we randomly selected 12 cases from the pre-implementation period and 12 cases from the post-implementation period. No eligible cases meeting inclusion criteria were excluded during the selection process. These five departments were selected because they accounted for 86.3% of the hospital's elective operating room (OR) volume and represented diverse procedural workflows, thereby ensuring the generalizability of findings across surgical specialties.

Inclusion criteria:

1. scheduled weekday cases during regular working hours;
2. non-first cases of the day in the OR;
3. patients aged ≥18 years.

Exclusion criteria:

1. first case of the day;
2. emergency surgeries;
3. ambulatory (same-day discharge) procedures;
4. cases involving patients with infectious diseases.

Baseline demographic and clinical characteristics were extracted from routine perioperative records. The following variables were selected *a priori* based on their potential influence on turnover time: sex, age, body mass index (BMI), ASA Physical Status Classification (I–III), presence of comorbidities (e.g., hypertension, diabetes, coronary artery disease), surgical specialty(general surgery, orthopedics, obstetrics and gynecology, urology, thoracic surgery), operating room class(Class I or II, reflecting cleanliness level per Chinese standards), and anesthesia type(endotracheal general, intravenous, or neuraxial anesthesia).

### Sample size justification

Based on a pilot analysis of 20 cases (10 pre- and 10 post-implementation), the mean turnover time was 54.2 ± 9.1 min pre-implementation and 44.5 ± 8.6 min post-implementation (Cohen's d = 1.09). To detect a 10-minute difference with 80% power at a two-sided *α* = 0.05, a minimum of 34 cases per group would have been required (calculated using G*Power 3.1). We enrolled 60 cases per group (12 per department) to account for subgroup analyses and potential data loss.

### Post hoc power analysis

Using the observed effect size in the full sample (Cohen's d = 1.45 for turnover time) showed >99% power for the primary outcome. For secondary outcomes, achieved power ranged from 72% (patient satisfaction) to 98% (surgeon satisfaction). We acknowledge that the study may be underpowered for certain subgroup comparisons and for detecting smaller effect sizes in secondary outcomes.

### Description of the exposure (management protocol)

**Figure 1:**
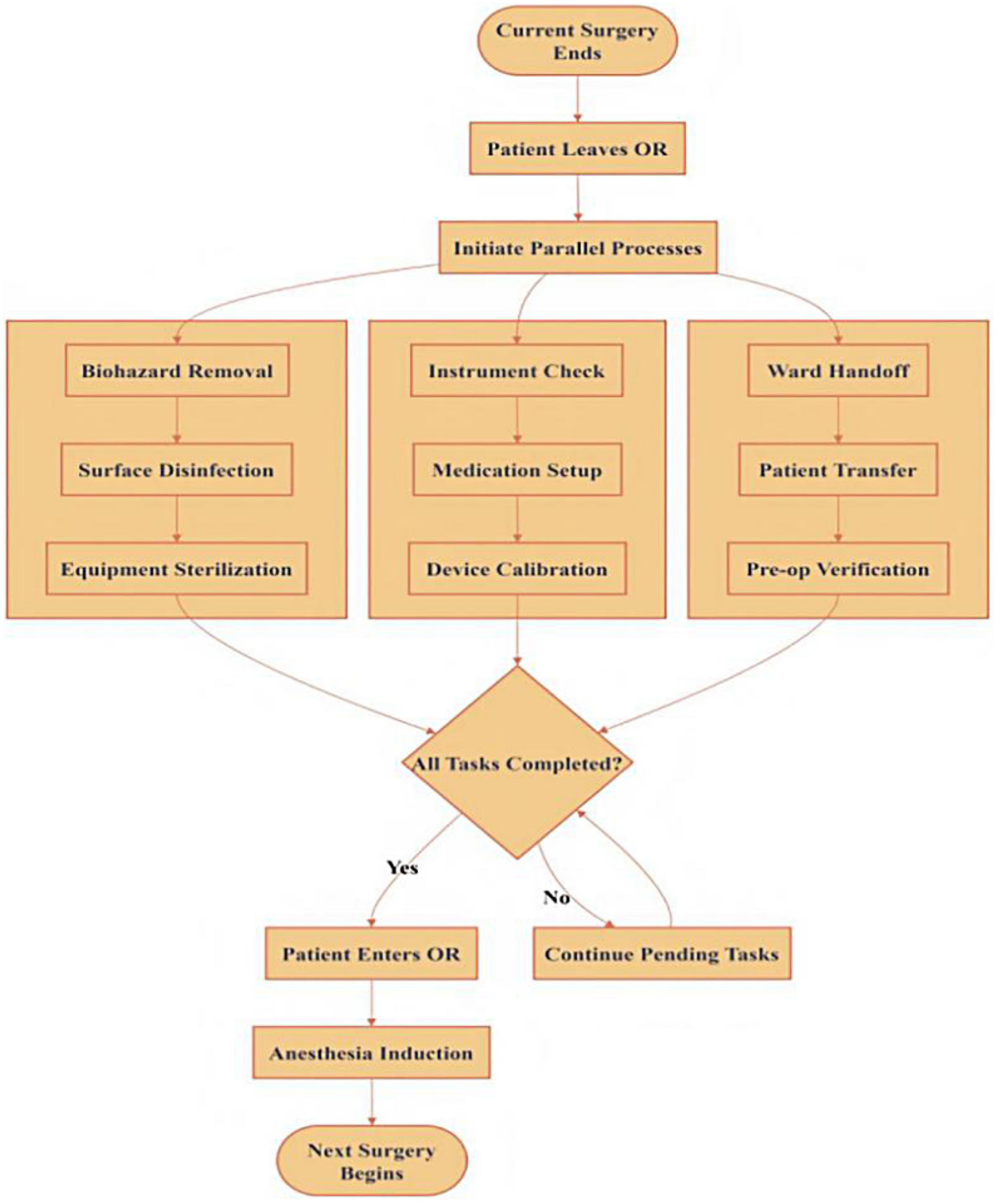
Optimized surgical turnover process workflow.

The management protocol introduced by hospital administration on August 1, 2024, comprised three key components:

1. Workflow redesign: Parallel task execution was introduced (e.g., patient transport initiated 30 min before estimated prior case end). Prior studies have demonstrated that VSM-based reengineering of surgical workflows effectively reduces idle time and improves operational efficiency, making it widely applicable for diagnosing inefficiencies in perioperative processes (12). Dedicated coordination roles were established, including an Infusion Coordination Nurse(“Jing Dian Ban”) responsible for real-time patient logistics and a Preoperative Assessment Nurse who conducted standardized preoperative visits using the MIPrep questionnaire, a validated, patient-reported measure of preoperative preparedness that emphasizes the patient's self-evaluation of readiness (13). Evidence indicates that enhanced patient-perceived preparedness is associated with improved surgical outcomes and perioperative experience (14). A 10-minute target for room cleaning and disinfection was set (Figure 1).
2. Information technology integration: The Medison Operating Room Information System was integrated for automatic timestamping (skin closure to skin incision). Electronic dashboards and WeChat (developed by Tencent Holdings Ltd., Shenzhen, China)-based interdepartmental communication groups were introduced for dynamic scheduling. Patient handover documentation was transitioned to a handheld personal digital assistant (PDA) device with barcode scanning. Some studies indicate that, the use of PDA enabled real-time access to updated surgical schedules and patient information, reducing communication delays and coordination cost s (15).
3. Team-based training: Monthly cross-functional simulation drills were implemented. Standardized daily briefings at 07:50 were introduced. A recognition program for high-performing teams was established.

## Data collection and outcome measures

### Primary efficiency indicators

Turnover time: Defined as the time interval (in minutes) from the completion of skin closure of the preceding surgical case to the start of skin incision for the subsequent case (16), automatically recorded by the Medison system and cross-verified against anesthesia and nursing records.

OR utilization rate: Calculated as (actual surgical duration/scheduled operating time) × 100 % (17). Scheduled operating time was standardized at 8 h per day. This indicator reflects the efficiency of operating room scheduling and time utilization.

### Quality and satisfaction indicators

Preoperative preparedness: Assessed using the MIPrep questionnaire (18), a validated patient-reported instrument consisting of 24 items. The scale demonstrated good internal consistency, with a Cronbach's *α* ranging from 0.81 to 0.89. Total scores were transformed to a 0–100 scale, with higher scores indicating greater patient readiness for surgery.

Surgeon satisfaction: Measured using a 5-point Likert scale (11) (1 = very dissatisfied, 5 = very satisfied), comprising eight items evaluating aspects such as workflow efficiency, team coordination, and scheduling reliability. The total score ranged from 8 to 40, with higher values reflecting greater satisfaction.

Patient satisfaction: Evaluated via a structured telephone interview conducted within 24 h post-discharge. The questionnaire included five items assessing key domains such as waiting time, staff attitude, and communication clarity, scored on a 0–10 numeric rating scale for each item.

### Process time decomposition

Four sub-phases were retrospectively extracted from routine operating room records and cross-verified with system logs: (1) patient transfer time (prior case end → next patient enters OR); (2) cleaning and disinfection time (patient exit → room ready); (3) instrument preparation time (tray release → setup complete); (4) anesthesia setup time (patient entry → anesthesia ready). Data from the pre-implementation period (February–July 2024, *n* = 60) and post-implementation period (August 2024–February 2025, *n* = 60) were retrieved from existing hospital databases. All time measurements were independently verified by two reviewers through dual data entry.

Patient satisfaction scores were obtained from routinely collected post-discharge telephone interviews conducted by the hospital quality department within 24 h of discharge. Surgeon satisfaction scores were extracted from monthly internal surveys routinely administered to attending surgeons. Preoperative preparedness (MIPrep) had been administered by Preoperative Assessment Nurse during preoperative visits as part of routine care; these scores were retrospectively retrieved and analyzed for this study. No additional data collection was performed by the research team.

### Statistical analysis

A retrospective before-after design was used in this study, comparing two historical cohorts defined by a hospital-wide policy change. Continuous variables—including turnover time, operating room utilization rate, preoperative preparedness, and satisfaction scores—were first assessed for normality using the Shapiro–Wilk test. Normally distributed data were summarized as mean ± standard deviation (Mean ± SD) and compared between groups using the independent samples t-test. For variables that violated the assumption of normality (patient satisfaction and operating room utilization), the Mann–Whitney U test (rank-sum test) was employed.

Categorical variables (e.g., sex, ASA physical status classification, departmental distribution) were presented as frequency (percentage). Between-group comparisons were performed using the *χ*^2^ test or Fisher's exact test, as appropriate (the latter applied when expected cell counts were <5).

The reduction rate for each component of turnover time was calculated as:

$$\begin{array}{r} \mathrm{Reduction}\;\mathrm{rate}(\text{\%})=[(\mathrm{pre}\text{-}\mathrm{implementation}\;\mathrm{time}\\ \;-\mathrm{post}\text{-}\mathrm{implementation}\;\mathrm{time})/\mathrm{pre}\text{-}\mathrm{implementation}\;\mathrm{time}]\times 100\text{\%}. \end{array}$$

Bivariate associations between turnover time and satisfaction scores were assessed using Pearson correlation (r).

All statistical tests were two-sided, with a significance level set at *α* = 0.05. Exact *P* values are reported to three decimal places; values below 0.001 are denoted as *P* < 0.001. All 95% confidence intervals (CIs) are presented as “lower bound to upper bound” with three decimal places. Data analysis was conducted using SPSS software (version 21.0; IBM, Armonk, NY, USA).

## Results

### Baseline characteristics of study participants

As specified in the Methods section, a total of 120 surgical cases were included in this study, with 60 cases in the pre-implementation group and 60 in the post-implementation group. No statistically significant differences were observed between the two groups in baseline characteristics.

There were no differences in sex distribution (male: 53.7% vs. 46.3%, *P* = 0.463), age (60.80 ± 13.67 vs. 56.31 ± 16.75 years, *P* = 0.243), or BMI (24.56 ± 3.63 vs. 24.88 ± 3.72, *P* = 0.269). Similarly, no significant differences were found in ASA physical status classification (*P* = 0.491), comorbidity profile (*P* = 0.244), distribution across surgical specialties (general surgery, orthopedics, etc., 12 cases per department in each group, *P* = 1.00), operating room class (*P* = 0.667), or anesthesia type (endotracheal general anesthesia: 84.2% vs. 85.0%, *P* = 0.784).

All comparisons yielded *P* > 0.05, indicating good baseline comparability between groups. (Table 1).

**Table 1:** Baseline characteristics of study participants.

Variable	Pre-implementation Group, *n* (%)	Post-implementation Group, *n* (%)	*P* value
Sex, no. (%)			0.463
Male	29 (48.3)	25 (41.7)	
Female	31 (51.7)	35 (58.3)	
Age (years), mean ± SD	60.80 ± 13.67	56.31 ± 16.75	0.243
BMI, mean ± SD	24.56 ± 3.63	24.88 ± 3.72	0.269
BMI category, no. (%)			
18.5–25	38 (53.5)	33 (46.5)	
25–30	19 (50.0)	19 (50.0)	
30–35	3 (27.3)	8 (72.7)	
ASA Physical Status, no. (%)			0.491
I	1 (100.0)	0 (0.0)	
II	37 (47.4)	41 (52.6)	
III	22 (53.7)	19 (46.3)	
Comorbidities, no. (%)			0.244
Diabetes	2 (100.0)	0 (0.0)	
Hypertension	3 (33.3)	6 (66.7)	
Coronary artery disease	1 (33.3)	2 (66.7)	
Other	54 (50.9)	52 (49.1)	
Department, no. (%)			1.00
General surgery	12 (50.0)	12 (50.0)	
Orthopedics	12 (50.0)	12 (50.0)	
Obstetrics and gynecology	12 (50.0)	12 (50.0)	
Urology	12 (50.0)	12 (50.0)	
Thoracic surgery	12 (50.0)	12 (50.0)	
Operating Room Class, no. (%)			0.667
Class I	13 (46.4)	15 (53.6)	
Class II	47 (51.1)	45 (48.9)	
Anesthesia Type, no. (%)			0.784
Endotracheal general anesthesia	50 (49.5)	51 (50.5)	
Intravenous anesthesia	3 (42.9)	4 (57.1)	
Neuraxial anesthesia	7 (58.3)	5 (41.7)	

BMI, Body Mass Index.

ASA: ASA Physical Status Classification.

Data are expressed as mean ± standard deviation, or numbers.

### Operating room efficiency outcomes

**Figure 2:**
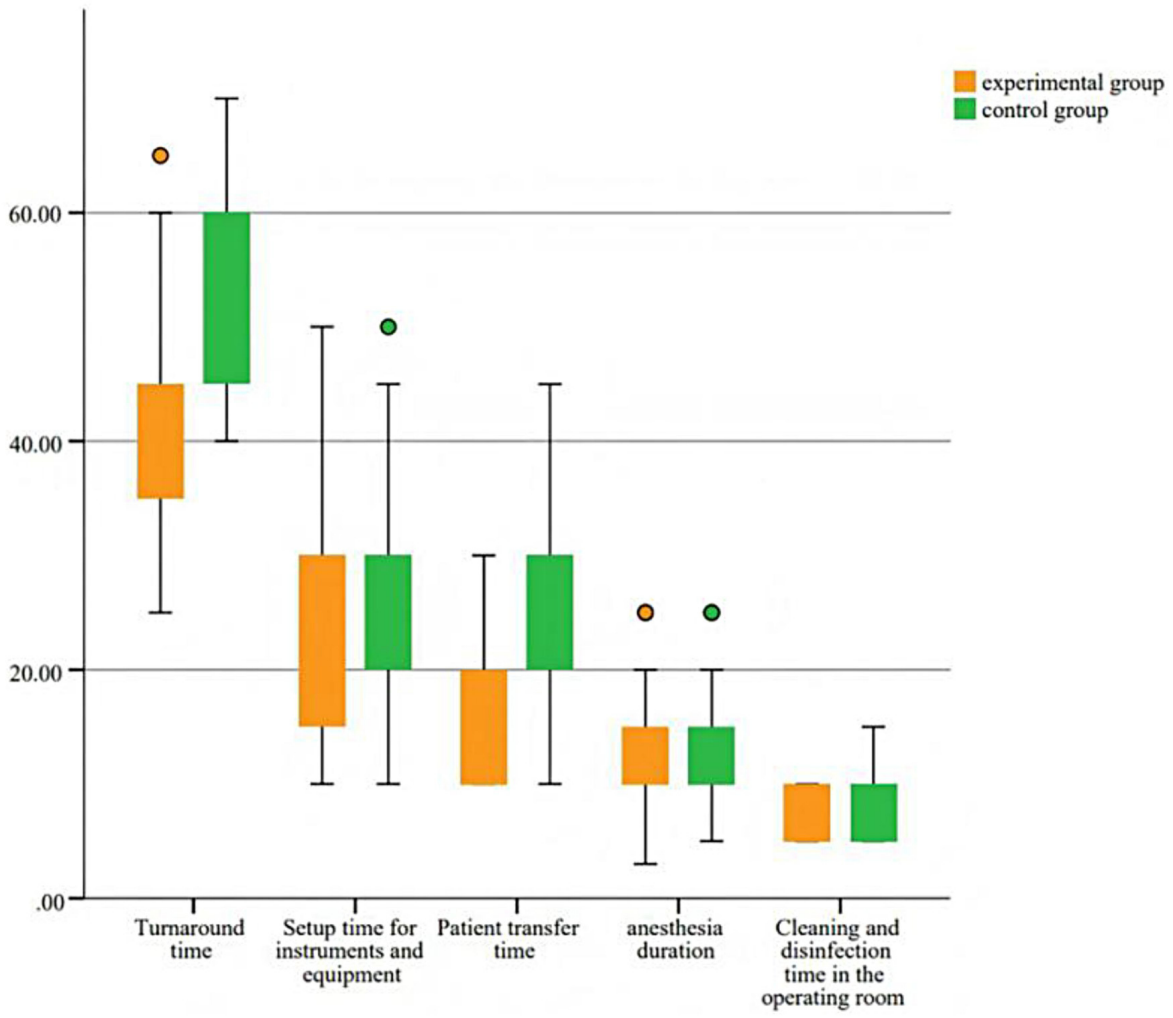
Boxplot of surgical turnover times before and after implementation of the management strategy.

The post-implementation cohort showed a shorter turnover time compared with the pre-implementation cohort (40.75 ± 8.33 vs. 52.75 ± 8.20 min; *Δ* = –12.00 min, 95% CI: −14.988 to −9.011; *P* = 0.008), representing a 22.7% difference. Operating room utilization was also higher in the post-implementation cohort (82.33% ± 20.78% vs. 71.91% ± 25.35%; *Δ* =  + 10.42 percentage points, 95% CI: 2.034 to 18.798; *P* = 0.037) (Table 2, Figure 2).

**Table 2:** Comparison of operating room operational efficiency before and after implementation of the management strategy.

Observation Indicator	Pre-implementation Group	Post-implementation Group	Mean Difference (95% CI)	*P* Value
Turnover time (minutes)	52.75 ± 8.20	40.75 ± 8.33	−12.00 (95% CI: −14.988 to −9.011)	0.008
Operating room utilization (%)	71.91 ± 25.35	82.33 ± 20.78	10.42 (95% CI: 2.034 to 18.798)	0.037

Data are expressed as mean ± standard deviation, or numbers.

### Differences in turnover process phases between cohorts

**Figure 3:**
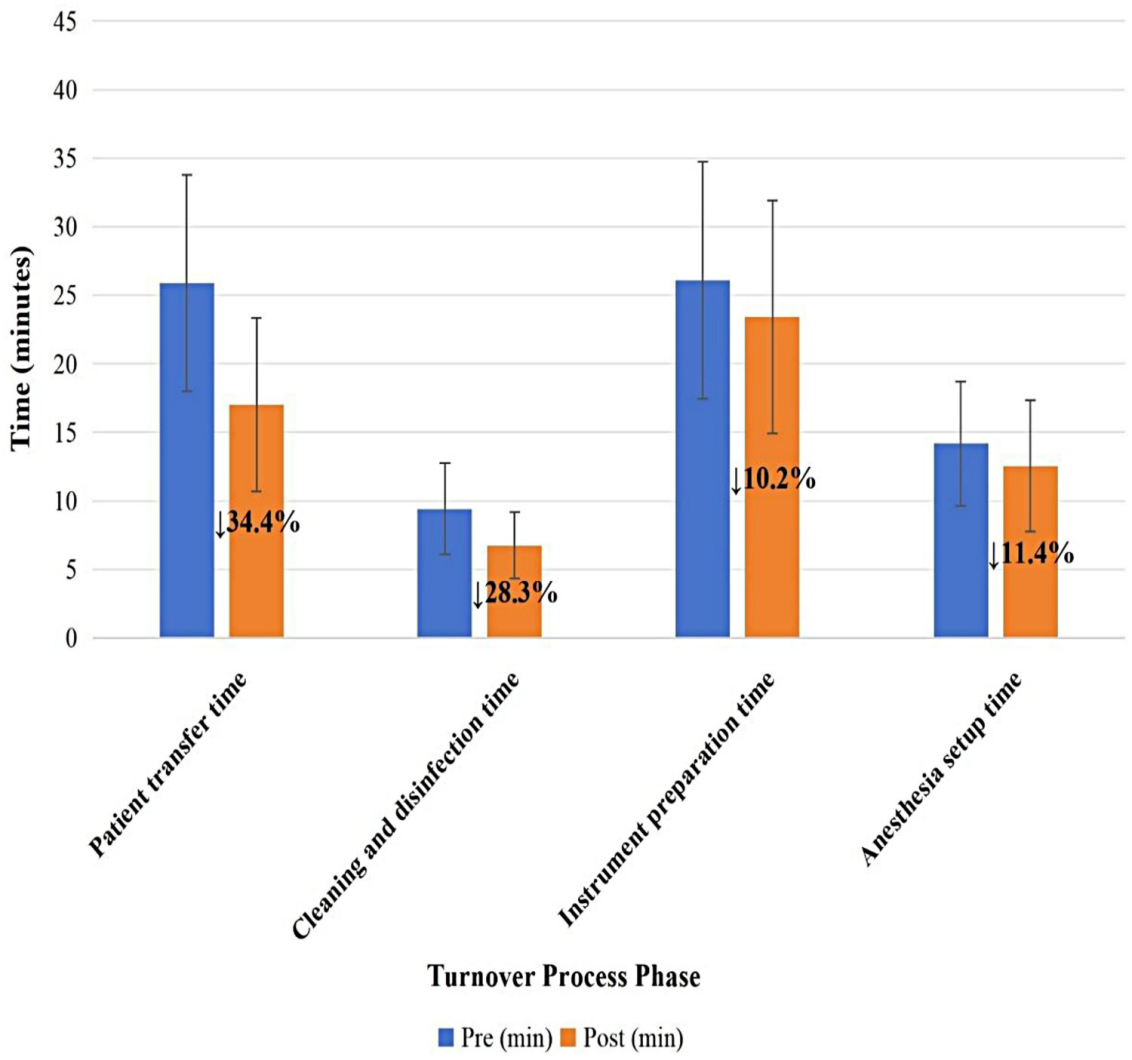
Comparison of turnover process phase times between pre-implementation and post-implementation cohorts. Bars represent mean time (minutes); error bars indicate standard deviation. Percentages above post-implementation bars indicate the percent difference relative to pre-implementation values.

Analysis of individual turnover phases revealed differences between the pre- and post-implementation cohorts (Figure 3). The sum of individual phase times exceeded the total measured turnover time—75.57 vs. 52.75 min pre-implementation, and 59.72 vs. 40.75 min post-implementation—reflecting overlapping and parallel execution of tasks under the new protocol (see Supplementary Table S1 for detailed component data).

The largest difference was observed in patient transfer time, which was 34.4% lower in the post-implementation cohort (25.91 ± 7.89 vs. 17.00 ± 6.32 min; *Δ* = –8.91 min, 95% CI: −11.502 to −6.331, *P* < 0.001), accounting for 74.3% of the total time difference.

Cleaning and disinfection time was also lower in the post-implementation cohort (9.41 ± 3.33 vs. 6.75 ± 2.40 min; *Δ* = –2.67 min, 95% CI: −3.717 to −1.616, *P* < 0.001), representing a 28.3% difference.

Smaller differences were observed in instrument preparation time (26.08 ± 8.64 vs. 23.42 ± 8.51 min; *Δ* = –2.67 min, 95% CI: −5.767 to 0.434, *P* = 0.041) and anesthesia setup time (14.17 ± 4.52 vs. 12.55 ± 4.79 min; *Δ* = –1.62 min, 95% CI: −3.303 to 0.069, *P* = 0.028), representing differences of 10.2% and 11.4%, respectively.

The magnitude of the observed differences followed a gradient: patient transfer > cleaning > instrument preparation > anesthesia (Figure 3).

### Differences in care quality and satisfaction between cohorts

The post-implementation cohort showed higher scores on all quality and satisfaction measures compared with the pre-implementation cohort (Table 3).

**Table 3:** Patient preparedness and satisfaction outcomes.

Outcome Measure	Pre-implementation Group	Post-implementation Group	Mean Difference (95% CI)	*P* Value
Preoperative preparedness (MIPrep total score)	81.38 ± 4.69	87.61 ± 6.22	6.233 (4.239 to 8.227)	0.023
Patient satisfaction (composite score)	35.32 ± 2.42	39.05 ± 2.42	3.7 (2.824 to 4.576)	0.005
Surgeon satisfaction (composite score)	26.03 ± 2.41	31.42 ± 3.08	5.383 (4.382 to 6.384)	0.017

Data are expressed as mean ± standard deviation, or numbers.

Preoperative preparedness, assessed using the MIPrep questionnaire, was higher in the post-implementation cohort (87.61 ± 6.22 vs. 81.38 ± 4.69; *Δ* =  + 6.23 points, 95% CI: 4.24 to 8.23, *P* = 0.023), representing a 7.7% difference.

Patient satisfaction (composite score across five domains, range 0–50) was higher in the post-implementation cohort (39.05 ± 2.42 vs. 35.32 ± 2.42; *Δ* =  + 3.73 points, 95% CI: 2.82 to 4.58, *P* = 0.005), representing a 10.6% difference. A negative correlation was observed between turnover time and patient satisfaction (r = –0.72, 95% CI: −0.84 to −0.55, *P* = 0.008).

Surgeon satisfaction (composite score across eight domains, range 8–40) was higher in the post-implementation cohort (31.42 ± 3.08 vs. 26.03 ± 2.41; *Δ* =  + 5.39 points, 95% CI: 4.38 to 6.38, *P* = 0.017), representing a 20.7% difference.

## Discussion

In this retrospective before-after study, the post-implementation cohort showed a shorter turnover time (*Δ* = –12.00 min, 22.7%), higher operating room utilization (*Δ* =  + 10.42 percentage points), and higher scores for preoperative preparedness, patient satisfaction, and surgeon satisfaction compared with the pre-implementation cohort. These observed differences are consistent with the hypothesis that the hospital's new management protocol may have contributed to operational improvements. However, because the study lacked a concurrent control group and used a non-randomized, historical cohort design, causal inference is limited. All findings should be interpreted as associations rather than as proven causal effects.

The magnitude of the observed turnover time difference is comparable to international reports. Hedden et al. reported a 19-minute reduction in turnover time for joint replacement surgery following a dedicated efficiency model (19). Similarly, Reeves et al. demonstrated that implementing efficiency metrics in an ambulatory surgery center was associated with improved schedule adherence and reduced turnover delays (20). Our findings extend this evidence by showing similar patterns across multiple surgical specialties (general surgery, orthopedics, obstetrics and gynecology, urology, and thoracic surgery), suggesting that the observed associations may be generalizable to a range of elective non-first surgeries in tertiary hospital settings.

The largest difference between cohorts was observed in patient transfer time, which was 34.4% lower in the post-implementation cohort and accounted for 74.3% of the total time difference. This finding is consistent with lean management principles that emphasize eliminating non-value-added waiting time (21). The 28.3% difference in cleaning and disinfection time aligns with evidence that parallel processing can optimize secondary workflows (22). Furthermore, computational algorithms for surgical scheduling have been shown to enhance team dynamics and reduce idle time between cases, supporting the integration of information technology into perioperative workflow redesign (23). Similarly, Sarpong et al. demonstrated that assigning consecutive cases to the same surgeon and anesthesia team was associated with shorter turnover times, highlighting the importance of team continuity in perioperative efficiency (24). The overlap/waiting time decreased significantly from 22.82 ± 6.18 min pre-implementation to 18.97 ± 3.69 min post-implementation (*Δ* = –3.85 min, *P* = 0.006), reflecting a shift from serial to parallel task execution under the new protocol. Instrument preparation time, although showing a smaller difference (10.2%), still accounted for 49.4% of total turnover time in the post-implementation cohort, indicating that supply chain and specialty-specific instrument availability remain important areas for further improvement.

The post-implementation cohort also showed higher preoperative preparedness scores (*Δ* =  + 6.23 points, 7.7%). This finding aligns with evidence from a randomized trial by Sassani et al., which demonstrated that structured preoperative counseling significantly improves patients’ perceived readiness for surgery (25).The observed differences in patient satisfaction (*Δ* =  + 3.73 points, 10.6%) and surgeon satisfaction (*Δ* =  + 5.39 points, 20.7%) are consistent with the hypothesis that workflow improvements may be associated with reduced patient waiting anxiety (26) and with alleviated provider communication burden (27). Furthermore, a systematic review by Rathnayake et al. confirmed that reducing waiting times is a key strategy for improving patient access to elective surgery and enhancing overall satisfaction (28). The negative correlation between turnover time and patient satisfaction (r = –0.72) further supports the notion that shorter waiting periods are associated with better patient-reported experiences.

### Study limitations

Several important limitations should be acknowledged.

First, this study employed a retrospective before-after design without a concurrent control group or randomization. Assignment to cohorts was determined solely by surgery date relative to a hospital policy change. Consequently, we cannot rule out the influence of secular trends (e.g., seasonal variation in surgical volume, gradual staff acclimatization to workflows, or concurrent institutional changes) or regression to the mean. A *post-hoc* interrupted time series analysis was not performed, as the limited number of monthly data points (6 pre-implementation and 6 post-implementation) would have yielded unreliable estimates; therefore, the possibility that pre-existing downward trends contributed to the observed differences cannot be excluded. All findings should be interpreted as associations, not causal effects.

Second, the exposure was a multicomponent bundle introduced simultaneously. The protocol included workflow redesign, new coordination roles, information technology integration, training, dashboards, drills, briefings, and feedback monitoring. Because all components were implemented together, we cannot isolate the effect of any single element. The observed differences should be attributed to the combined package rather than to any individual component. Language that attributed effects to specific roles (e.g., the Infusion Coordination Nurse alone) has been removed from this revised manuscript.

Third, several unmeasured or partially measured confounders may influence turnover time, including procedure complexity (minor vs. major surgery within the same specialty), case duration, day of week, staffing levels by shift, surgeon and team experience, and specialty-specific instrument burden. While we balanced measured baseline characteristics between cohorts, residual confounding cannot be excluded. We do not rely on non-significant baseline *P* values as proof of comparability.

Fourth, the presence of trained observers who manually timed process steps and a central quality control team that provided weekly feedback may have influenced staff behavior independently of the protocol content (Hawthorne effect). Effects observed under active study monitoring may differ from routine practice without such oversight. We did not formally assess inter-rater reliability for manual time measurements.

Fifth, the sample size was modest and limited to a single institution, which may restrict external validity. The *post hoc* power analysis showed >99% power for the primary outcome but only 72% power for patient satisfaction, indicating a 28% risk of type II error for this measure. The study may be underpowered for certain subgroup comparisons and for detecting smaller effect sizes in secondary outcomes.

Sixth, cases within the same department, operating room, or surgical team are unlikely to be independent observations. Our analysis treated each case as independent, which may underestimate standard errors. We did not employ mixed-effects models or cluster-robust standard errors.

Seventh, these findings are most directly applicable to adult, elective, non-first surgeries in tertiary hospital settings similar to our institution. Generalizability to other settings (e.g., primary hospitals, emergency surgeries, ambulatory procedures, or different healthcare systems) requires further validation.

### Future directions

Beyond the parallel task processing implemented in the present protocol, several promising strategies warrant further investigation. Overlapping surgery models, where a single surgeon alternates between two operating rooms, have been shown to reduce between-case idle time and increase surgeon throughput in high-volume specialties. Additionally, dedicated anesthetizing rooms (induction rooms) adjacent to operating rooms can significantly reduce post-operative anesthesia setup time. Our institution is currently evaluating the feasibility of these models. Future research should employ prospective controlled designs, such as stepped-wedge cluster randomized trials or matched-pair hospital comparisons, to strengthen causal inference. Larger multicenter studies with longer follow-up are needed to confirm the generalizability of the observed associations and to assess the sustainability of the improvements.

## Data Availability

The raw data supporting the conclusions of this article will be made available by the authors, without undue reservation.

## Supplementary material

Supplementary Table S1 Detailed turnover process phase components.
