# Supplementary material for: Association of an integrated management strategy with operating room efficiency, turnover time, and satisfaction: a retrospective before-after study
Source: Front Health Serv. 2026 Jun 26;6:1796885. doi: 10.3389/frhs.2026.1796885 (PMC13350319; doi:10.3389/frhs.2026.1796885)
Supplement: Supplementary Table S1 — Detailed turnover process phase components. [file Table1.docx]

| Supplementary Table S1. Detailed turnover process phase components | | | | |
| --- | --- | --- | --- | --- |
| Phase | Pre-implementation Group ( min) | Post-implementation Group ( min) | Mean Difference (95% CI) | *P* Value |
| Total turnover time | 52.75±8.20 | 40.75±8.33 | -12.00 (95% CI: -14.988 to -9.011) | ＜0.001 |
| Patient transfer time | 25.91±7.89 | 17.00±6.32 | -8.92 (95% CI: -11.502 to -6.331) | ＜0.001 |
| Cleaning and disinfection time | 9.41±3.33 | 6.75±2.40 | -2.67 (95% CI: -3.717 to -1.616) | ＜0.001 |
| Instrument preparation time | 26.08±8.64 | 23.42±8.51 | -2.67 (95% CI: -5.23 to -0.10) | 0.041 |
| Anesthesia setup time | 14.17±4.52 | 12.55±4.79 | -1.62 (95% CI: -3.18 to -0.06) | 0.028 |
| Sum of individual phases | 75.57±14.38 | 59.72±12.02 | -15.85 (95% CI: -20.59 to -11.11) | ＜0.001 |
| Overlap/waiting time | 22.82±6.18 | 18.97±3.69 | -3.85 (95% CI: -5.67 to -2.03) | 0.006 |
| Confidence intervals were recalculated using the exact degrees of freedom; minor rounding adjustments were made to ensure consistency with *P* values.  The total reception time = Σ sub-item time - overlapping time of links (post-implementation group) or + waiting time (pre-implementation group)  Data are expressed as mean ± standard deviation, or numbers | | | | |
